# Supplementary material for: Promoter-enhancer interactions identified from Hi-C data using probabilistic models and hierarchical topological domains
Source: Nat Commun. 2017 Dec 21;8:2237. doi: 10.1038/s41467-017-02386-3 (PMC5740158; doi:10.1038/s41467-017-02386-3)
Supplement: Supplementary file 1 — Supplementary Information [file 41467_2017_2386_MOESM1_ESM.pdf]

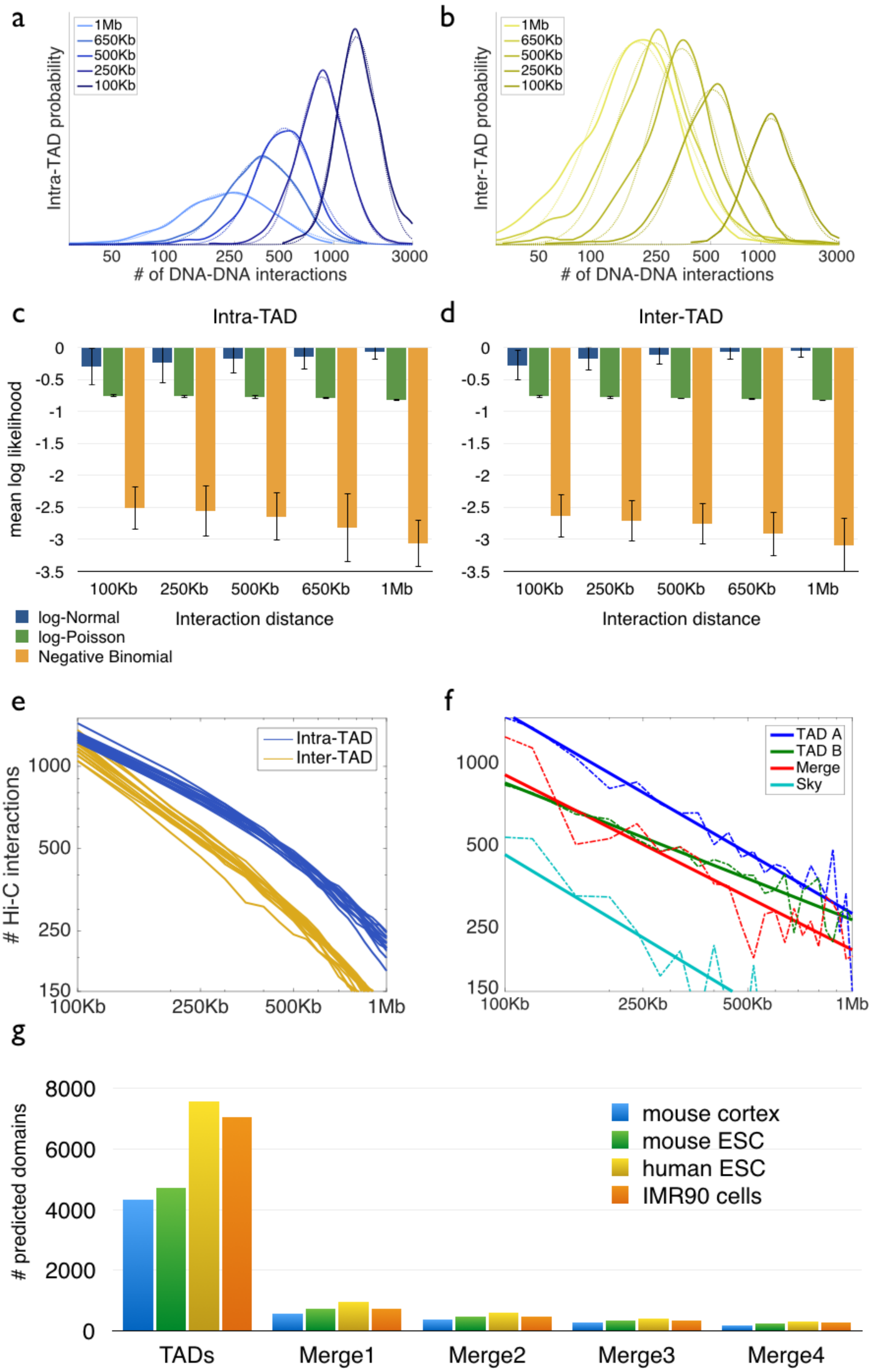
a
Intra-TAD probability
1Mb
650Kb
500Kb
250Kb
100Kb
# of DNA-DNA interactions
b
Inter-TAD probability
c
Intra-TAD
d
Inter-TAD
mean log likelihood
100Kb
250Kb
500Kb
650Kb
1Mb
Interaction distance
log-Normal
log-Poisson
Negative Binomial
e
Hi-C interactions
Intra-TAD
Inter-TAD
f
TAD A
TAD B
Merge
Sky
g
predicted domains
mouse cortex
mouse ESC
human ESC
IMR90 cells
TADs
Merge1
Merge2
Merge3
Merge4

Supplementary Figure 1. a-b. Solid lines show the histograms of Hi-C cells (number of DNA-DNA interactions) among intra-TAD (blue) and inter-TAD (yellow) pairs located 100Kb, 250Kb, 500Kb, 650Kb and 1Mb apart. Each histogram is fitted by a matched log-Normal distribution (shown as dotted lines). Theser results suggest that the number of Hi-C interactions, for multiple scales, can be accurately approximated by a log-Normal distribution. Analyzed are data from chr 11, mouse ES cells[25]. Distribution were normalized according to their matching *a priori* probabilities, resulting with increased probability for short-range pairs for the intra-TAD models, and long-range pairs for inter-TAD models. **c-d.** Average $\log_{10}$ likelihood of intra-TAD Hi-C counts (from A) and inter-TAD counts (from B) approximated using log-Normal distributions (blue), log-Poisson (green) or Negative-Binomial distribution (orange). These results support PSYCHIC's log-Normal models. **e.** Robustness of log-Normal parameter estimation, comparing estimated $\boldsymbol{\mu_d^{intra}}$ and $\boldsymbol{\mu_d^{inter}}$ values across different chromosomes **f.** Differences in power-law distributions for TADs A and B (as shown in Figure 1D), their merged interactions and the inter-TAD background interactions (denoted as "Sky"). Y-axis corresponds to number of Hi-C interactions at different distances (X-axis). **g.** A comparison of the number of TADs and hierarchical merges (1st, 2nd order, etc) for various Hi-C datasets in mouse (cortex, ESC) and human (ESC, IMR90) from Dixon et al[25].

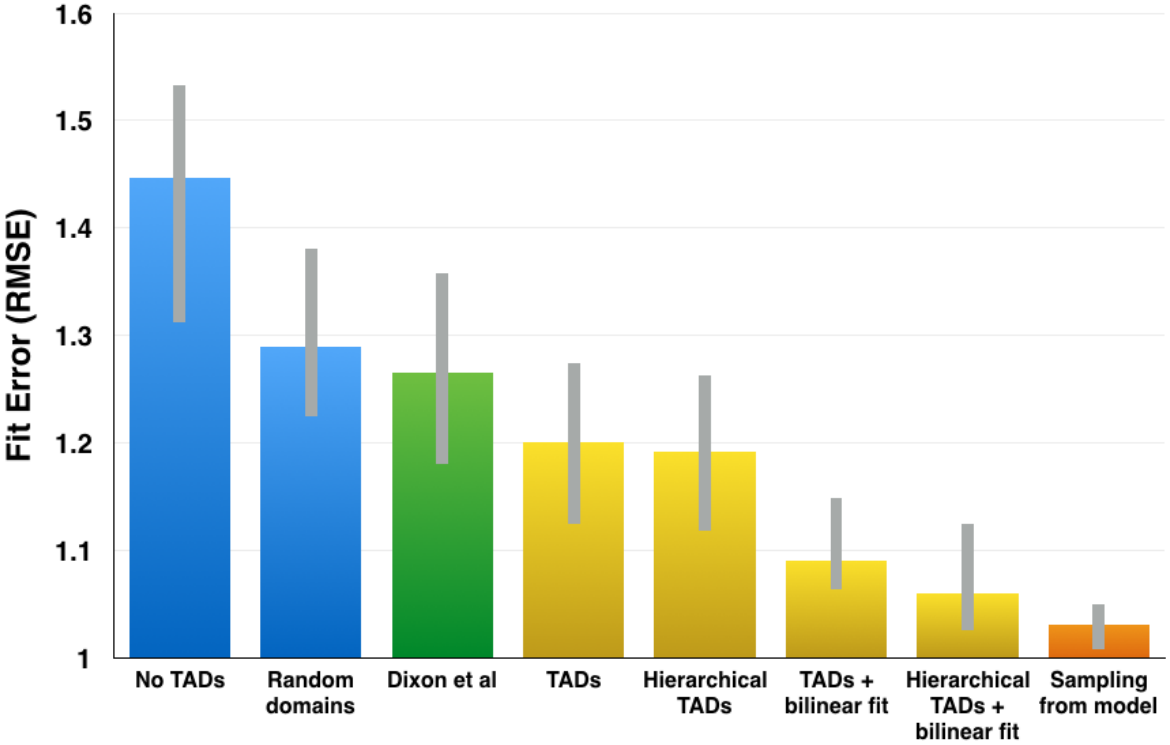

Supplementary Figure 2. PSYCHIC improves the modeling of Hi-C data using hierarchical TAD merges, and bilinear power-law fit. Shown are fit errors (root mean squared error, RMSE) of all Hi-C cells (up to 5Mb), averaged over all chromosomes in mouse cortex data[25]. Bars correspond to (from left to right): (1) fit for entire chromosome, with no TAD segmentation; (2) TAD-specific fit for random (shuffled) domains; (3) TAD-specific fit for Directionality Index domains from Dixon et al[25]; (4) TAD-specific fit for PSYCHIC-called domains; (5) fit for PSYCHIC domains with inter-TAD merges; (6) fit for PSYCHIC TADs with TAD-specific bi-linear fit model; (7) fit for hierarchical TADs with bi-linear fit models for TADs and merged regions; and (8) fit for synthetic data (simulated with similar sequencing read as real-life data) using the same TADs (and merges) that had generated it, to reflect the internal (sampling) noise in the data. RMSE values were also calculated for each chromosome, with error-bars reflecting the 25th and 75th percentiles.

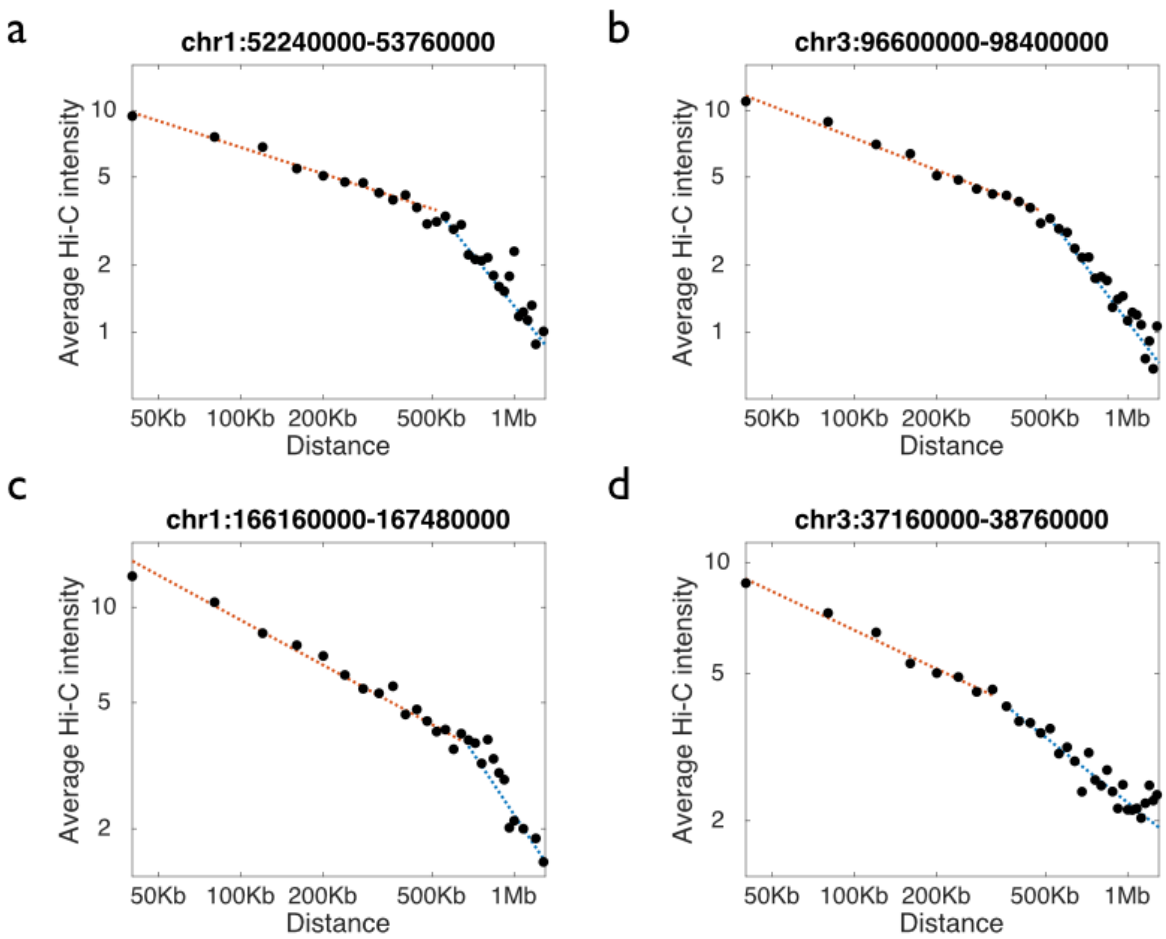

Supplementary Figure 3. TAD-specific bilinear power-law fit of Hi-C data, for four genomic loci using adult mouse Hi-C data[25]. Shown are the average numbers of Hi-C interactions (Y-axis) for each genomic distance between the interacting DNA loci (X-axis). Dotted lines mark the piecewise linear fit.

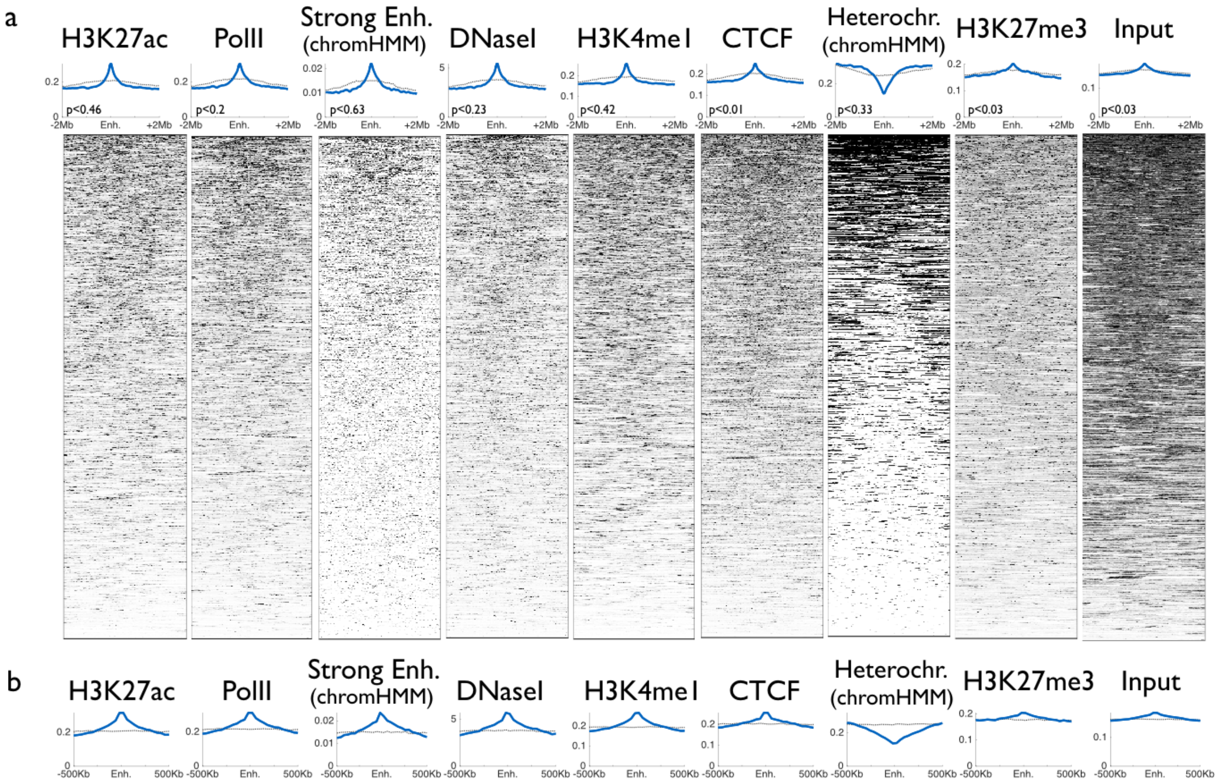

Supplementary Figure 4. a. Same as Figure 3, heatmaps showing random set of genomic loci, sampled in 2Mb windows around promoters (black dotted lines in the above plots). **b.** Same as Figure 3 (top) with 1Mb windows.

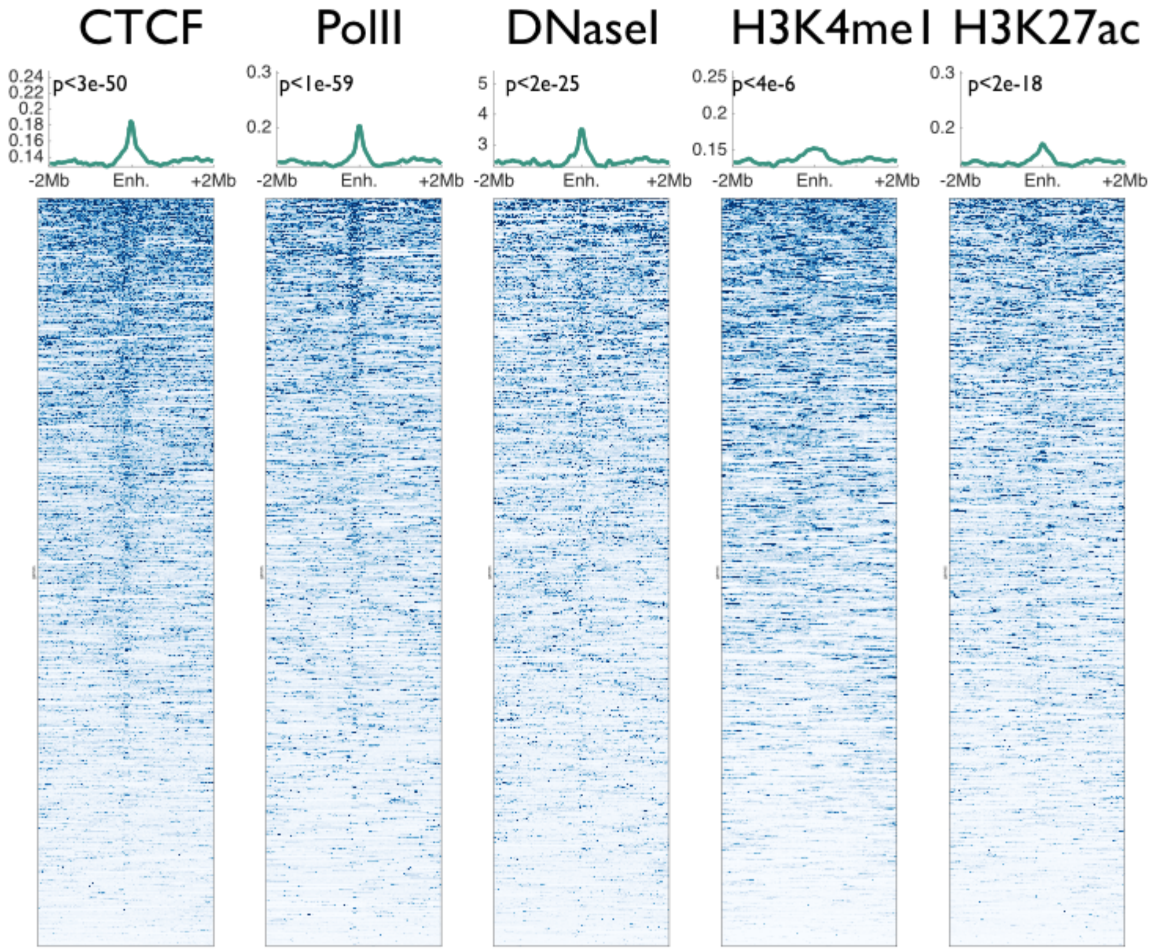

Supplementary Figure 5. Chromatin marks at 4Mb windows centered around TAD boundaries in mouse cortex[25], predicted by PSYCHIC. Shown are boundary mark (CTCF), transcription-related marks (PolII, H3K27ac), enhancer (H3K4me1) and DNaseI hypersensitivity assays. p-values as in Figure 3.

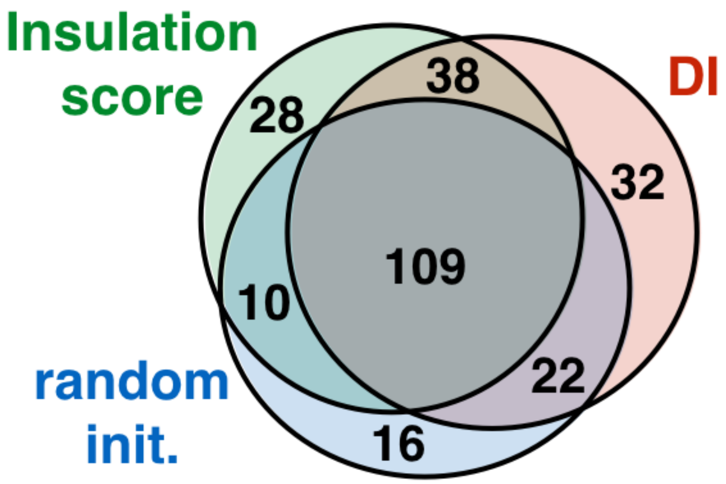

Supplementary Figure 6. Venn diagram plotting the effect of different initialization on the final set of predicted enhancer regions. PSYCHIC's two-component model was initialized by the Directionality Index method (DI, Dixon et al, 2012), Insulation Square Score (Crane et al, 2015), as well as initialization by random TADs. These changes had a limited effect on the predicted enhancer regions (called using an FDR threshold of 1e-4) on adult cortex Hi-C data (Dixon et al, 2012).

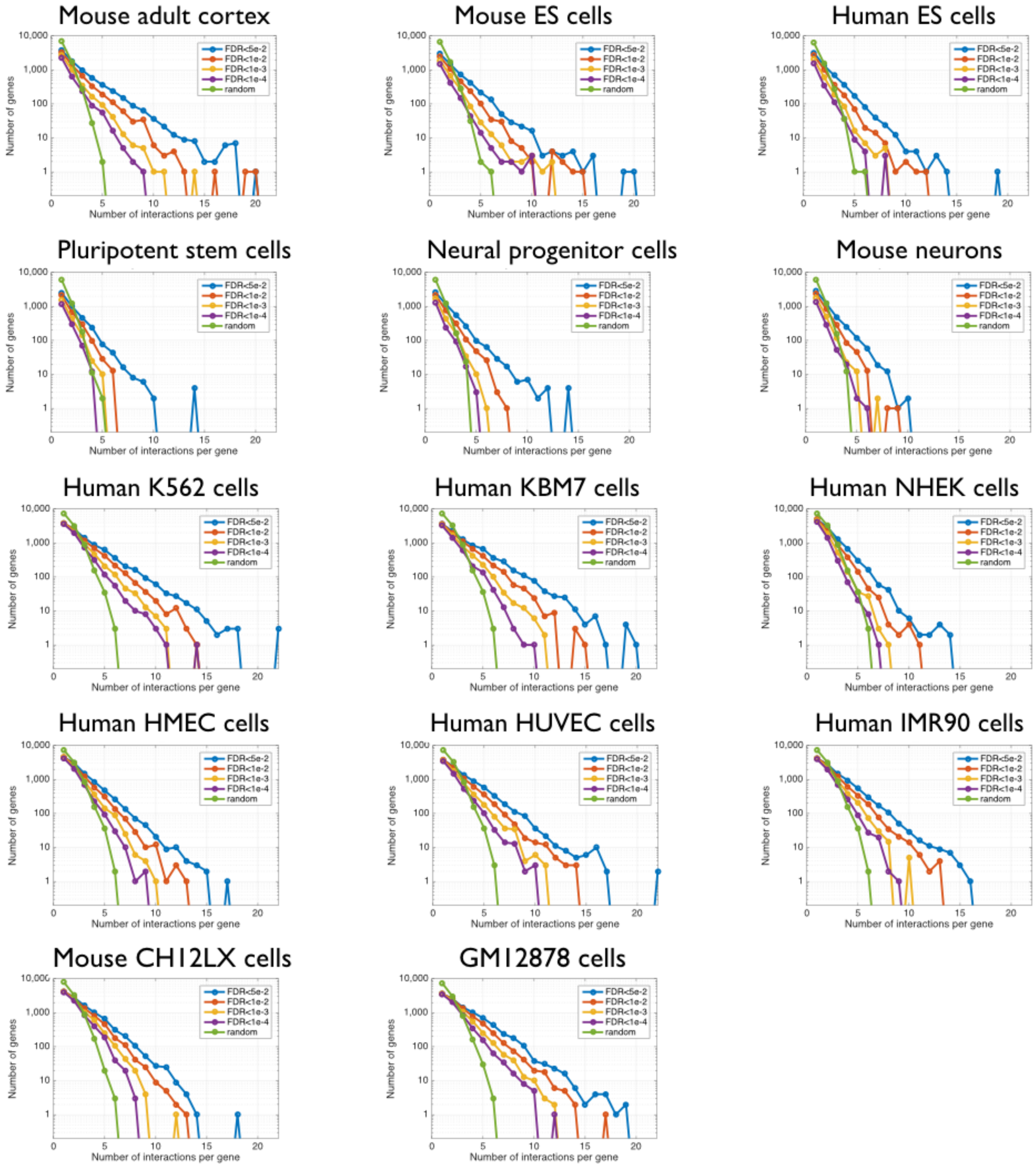

Supplementary Figure 7. Number of predicted enhancer regions per gene. For each Hi-C dataset, we ran PSYCHIC and predicted putative interactions for each promoter (up to a maximal distance of 1Mb), using several thresholds of statistical enrichment (FDR values of 0.05, 0.01, 1e-3 and 1e-4). Shown are the numbers of genes (Y-axis) predicted to be regulated by X putative enhancer regions (X-axis), compared to a random set of gene-surrounding genomic loci (in green, total size similar to the FDR<1e-2 set of putative enhancers).

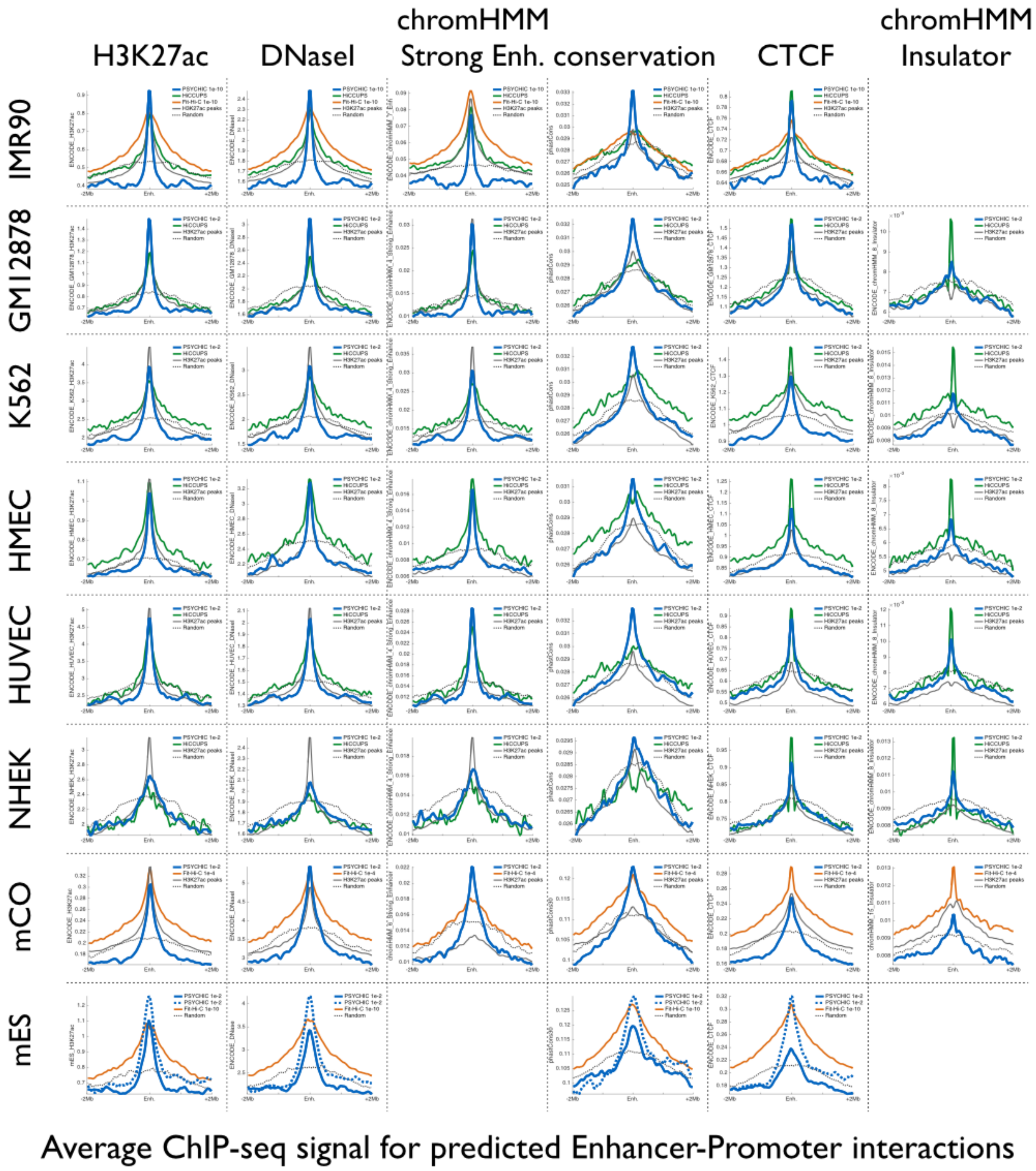

Supplementary Figure 8. Average signal for ChIP-seq and other genomic data around predicted enhancer regions, as in Figure 5. Shown are predictions by PSYCHIC (blue), HiCCUPS (Rao et al, 2014, plotted in green), Fit-Hi-C (Ay et al, 2014, plotted in orange), H3K27ac peaks, or random interactions (yellow). Notably, PSYCHIC predictions generally show stronger and sharper signal and for all enhancer-related data, while HiCCUPS and Fit-Hi-C predictions are more enriched for CTCF and Insulator marks.

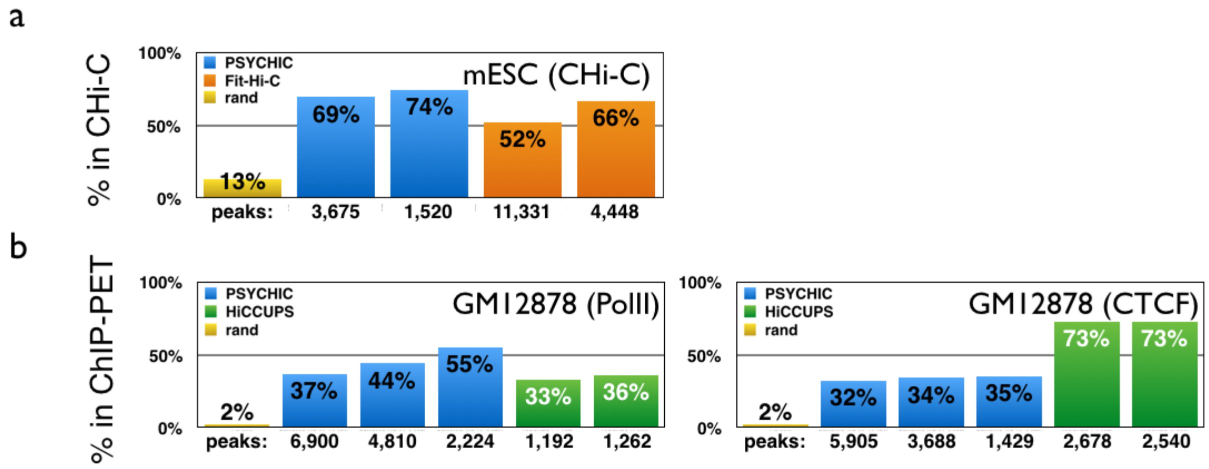

Supplementary Figure 9. Analysis of Capture Hi-C and ChIA-PET data. a. Similarly to Figure 6, we computed the percent of promoter-enhancer interactions (Y-axis) that are supported by Capture Hi-C data in mouse ES cells (Schoenfelder et al, 2015). These overlap of 69% of predicted interactions by PSYCHIC (called with an FDR threshold of p<1e-2), or 74% (PSYCHIC predictions with FDR<1e-4), compared to 52%-66% for Fit-Hi-C predictions (for mouse ES cells, Ay et al, 2014, thresholds of 1e-10 and 1e-20). **b.** Similar analysis for ChIA-PET data in GM12878 (Tang et al, 2015). Analysis of anti-PolII ChIA-PET interactions show high support for our predictions, ranging from 37% (PSYCHIC predictions in GM12878 with FDR threshold of 1e-2) to 55% (FDR<1e-10). HiCCUPS predictions for GM12878 (Rao et al, 2014) yielded lower coverage by PolII ChIP-PET peaks (33%-36%). Intriguingly, a much higher proportion of HiCCUPS calls (73%) were supported by CTCF ChIA-PET pairs, compared to only ~34% for PSYCHIC. This interesting result is in line with Figure 5, showing high ChIP-seq enrichments for CTCF (as well as Insulator marks) at putative enhancers called by HiCCUPS, and could be explained by HiCCUPS tendency to identify near-boundary interactions.

.

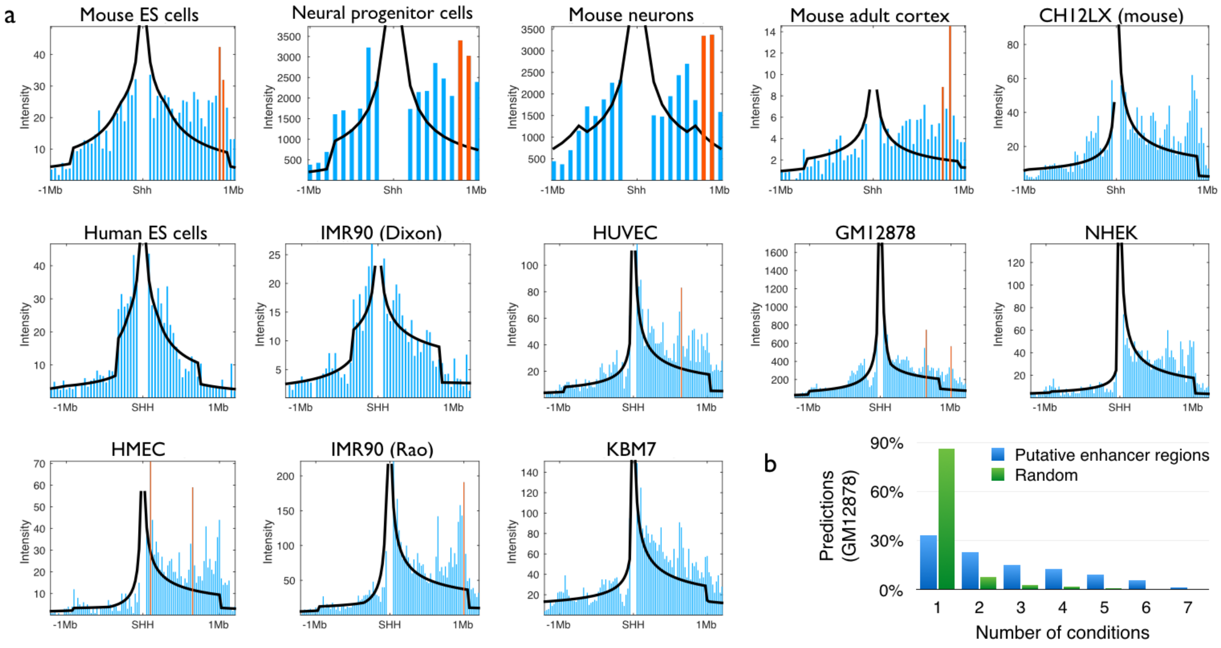

Supplementary Figure 10. a. Virtual 4C plots for Shh (Figure 7), in additional conditions in mouse and human. Interactions between Shh and the limb enhancer ZRS (located 850Kb away from Shh in mouse, 980Kb from the human SHH) are identified for most mouse Hi-C experiments, but not for the human Hi-C data, except for IMR90 and GM12878. **b.** Histogram comparing the percent of GM12878 putative enhancers (Y-axis) shared with other conditions (X-axis). Green bars correspond to random locations.

	PSYCHIC (FDR<1E-2)	PSYCHIC (FDR<1E-4)	PSYCHIC (FDR<1E-10)
**human ESC (Dixon et al)**	7,549	2,973	616
**IMR90 (Dixon et al)**	11,167	4,463	805
**GM12878 (Rao et al)**	28,822	16,791	6,291
**HMEC (Rao et al)**	26,838	16,016	6,279
**HUVEC (Rao et al)**	23,031	12,092	3,660
**IMR90 (Rao et al)**	24,541	14,219	5,086
**K562 (Rao et al)**	25,681	14,843	5,581
**KBM7 (Rao et al)**	15,225	7,571	2,178
**NHEK (Rao et al)**	16,891	9,075	2,763
**CH12LX (Rao et al)**	26,107	16,437	6,986
**mouse cortex (Dixon et al)**	17,788	7,088	1,567
**mouse ESC (Dixon et al)**	9,771	3,908	860
**pombo_ESC_HindIII (Fraser et al)**	5,344	1,881	299
**pombo_ESC_NcoI (Fraser et al)**	6,090	2,421	648
**pombo_Neuron_HindIII (Fraser et al)**	4,360	1,374	219
**pombo_Neuron_NcoI (Fraser et al)**	6,726	2,413	525
**pombo_NPC_HindIII (Fraser et al)**	5,670	1,923	223
**pombo_NPC_NcoI (Fraser et al)**	6,763	2,554	559

Supplementary Table 1. Number of predicted enhancer regions per experiment.
For each Hi-C dataset, we ran PSYCHIC and predicted putative interactions for each promoter (up to a maximal distance of 1Mb), using several thresholds of statistical enrichment (FDR values of 0.01, 1e-4 and 1e-10).
