# Supplementary material for: Promoter-enhancer interactions identified from Hi-C data using probabilistic models and hierarchical topological domains
Source: Nat Commun. 2017 Dec 21;8:2237. doi: 10.1038/s41467-017-02386-3 (PMC5740158; doi:10.1038/s41467-017-02386-3)
Supplement: Supplementary file 3 — Description of Additional Supplementary Files [file 41467_2017_2386_MOESM3_ESM.pdf]

Description of Additional Supplementary Files

File Name: Supplementary Data 1

Description: PSYCHIC predictions for human datasets (hg19). For each Hi-C dataset, we ran PSYCHIC and predicted putative interactions for each promoter (up to a maximal distance of 1Mb), using several thresholds of statistical enrichment (FDR values of 0.01, 1e-4 and 1e-10). These are found in a zip file containing one bed file (hg19) for each dataset. Also available at www.cs.huji.ac.il/~tommy/PSYCHIC/.

File Name: Supplementary Data 2

Description: PSYCHIC predictions for mouse datasets (mm9). For each Hi-C dataset, we ran PSYCHIC and predicted putative interactions for each promoter (up to a maximal distance of 1Mb), using several thresholds of statistical enrichment (FDR values of 0.01, 1e-4 and 1e-10). These are found in a zip file containing one bed file (mm9) for each dataset. Also available at www.cs.huji.ac.il/~tommy/PSYCHIC/.
